# Non-adherence to antidementia medications and associated factors: a study of Spanish population-based registry data

**DOI:** 10.3389/fphar.2024.1425442

**Published:** 2024-11-05

**Authors:** Eduardo Gutiérrez-Abejón, M. Aránzazu Pedrosa-Naudín, Diego Fernández-Lázaro, Isabel Díaz Planelles, F. Javier Álvarez

**Affiliations:** ^1^ Pharmacological Big Data Laboratory, Department of Cell Biology, Genetics, Histology and Pharmacology, Faculty of Medicine, University of Valladolid, Valladolid, Spain; ^2^ Valladolid Este Primary Care Department, Valladolid, Spain; ^3^ Pharmacy Directorate, Castilla y León Health Council, Valladolid, Spain; ^4^ Facultad de Empresa y Comunicación, Universidad Internacional de la Rioja (UNIR), Logroño, Spain; ^5^ Department of Cellular Biology, Genetics, Histology and Pharmacology, Faculty of Health Sciences, Campus of Soria, University of Valladolid, Soria, Spain; ^6^ Neurobiology Research Group, Faculty of Medicine, University of Valladolid, Valladolid, Spain; ^7^ CEIm of the Valladolid Health Areas, Valladolid, Spain

**Keywords:** dementia, medication adherence, drug utilization, cholinesterase inhibitors, memantine

## Abstract

**Introduction:**

With an increasing prevalence, dementia is one of the most disabling diseases among the elderly. Impaired cognitive function and behavioral and psychological symptoms predispose patients to medication non-adherence, resulting in increased morbidity, mortality, and healthcare costs. The aim of this study was to estimate the prevalence of non-adherence to antidementia medications and to identify the main predictors.

**Methods:**

A population-based registry study was conducted in 2022 in Castile and Leon, Spain. A total of 17,563 patients with dementia were included. The medication possession ratio (MPR) was used as an indirect method to measure adherence. The cut-off point for determining that a patient was nonadherent was 80% of MPR. Multivariate logistic regression was used to identify predictors of nonadherence based on sociodemographic and health-related variables.

**Results:**

In 2022, 6.2% of the population over 80 years old used antidementia medications. Of these patients, 70% were women, 28.15% were institutionalized, and over 90% were polymedicated and had multiple prescribers. The most used medicines were donepezil (43.49%), rivastigmine (36.84%), and memantine (30.7%). The combined use of an acetylcholinesterase inhibitor plus memantine was relevant (13.33%). Men were less adherent than women, and the prevalence of non-adherence decreased with age. The medication associated with the highest prevalence of non-adherence was rivastigmine (19%), followed by donepezil (17%) and memantine (13.23%). Institutionalized patients (13%) and patients on combination therapy (13.29%) had the lowest prevalence of non-adherence. Protective factors against non-adherence include institutionalization, polymedication, use of memantine or combination therapy, and comorbid mental illness.

**Conclusions:**

In Castile and Leon, one in six patients were non-adherent to antidementia medications. Younger male patients with cardiometabolic disease are more likely to be non-adherent to antidementia medications. On the other hand, institutionalization is a protective factor against non-adherence, but still 10% of nursing home patients are non-adherent to antidementia medications.

## 1 Introduction

By 2050, almost 140 million people worldwide are expected to have some form of dementia (Gauthier et al., 2022). In Spain, over 700,000 people are living with dementia, with a maximum prevalence of 39.2% in people over 90 years of age (Spanish Ministry of Health, 2019).

Dementia is a leading cause of death and disability (Matthews et al., 2019), accounting for 11.9% of years lived with disability due to chronic disease (Spanish Ministry of Health, 2019). In addition, dementia is a significant economic burden, with costs equivalent to 1.1% of global gross domestic product (GDP), potentially reaching $2 billion by 2030 (Patterson, 2018). Dementia is characterized by progressive cognitive deterioration (National Collaborating Centre for Mental Health (UK), 2007). In addition, more than 90% of people with dementia experience behavioral and psychological symptoms (BPSD), including motor disorders and changes in behavior, mood, thought, and perception (Ballard et al., 2008; Wu et al., 2016).

Traditional antidementia medications improve cognitive function and BPDS, but do not change the course of the disease. This treatment is based on two classes of medicines: acetylcholinesterase inhibitors or anticholinesterases (AChEIs), such as donepezil, rivastigmine, and galantamine, and N-methyl-D-aspartic acid receptor antagonists (NMDARs), such as memantine (Arvanitakis et al., 2019). AChEIs are recommended for the treatment of mild to moderate dementia (Hort et al., 2010), while memantine is indicated for moderate to severe dementia (Ngo and Holroyd-Leduc, 2015; Wong, 2016).

The use of antidementia medications is controversial because their efficacy is questionable, especially for long-term use (Gardette et al., 2014), and side effects are common (10%–25%), especially with AChEIs (Olazarán et al., 2013) As a result, France, withdrew public coverage of these drugs in 2018 (Livingston et al., 2020). However, AChEIs and memantine are financed in the rest of Europe, North America, and Australia (Rodda and Carter, 2012). Moreover, new therapeutic targets have been investigated to develop medicines that may modify the course of dementia. To date, the Food and Drug Administration (FDA) has approved two anti-amyloid beta (Aβ) protein monoclonal antibodies (mAbs): aducanumab and lecanemab (Buccellato et al., 2023; Peng et al., 2023).

Adults with chronic diseases are known to be 30%–50% non-adherent to prescribed medications (Naderi et al., 2012). Non-adherence is associated with increased morbidity and mortality (Iuga and McGuire, 2014) and a high economic burden (Ho et al., 2008; Gutiérrez-Abejón et al., 2023). In this sense, non-adherence rates of up to 70% have been observed in patients using AChEIs (Kröger et al., 2010).

Due to the symptoms of the disease, patients with dementia are predisposed to non-adherence (Campbell et al., 2012). Memory impairment, depression, functional limitations, comorbidities (hypertension, coronary heart disease, diabetes, or chronic obstructive pulmonary disease), complex medication dosing regimens, and a high incidence of side effects are all barriers to adherence (Connolly et al., 2011; Nieuwlaat et al., 2014). Other relevant factors such as patient perception, comprehension difficulties, and dependence on a caregiver for treatment administration and follow-up should not be overlooked (Gardette et al., 2014).

The highest rates of non-adherence to antidementia medications are associated with older women with high levels of cognitive impairment and low levels of education (Maxwell et al., 2014; Bohlken et al., 2015). Moreover, concomitant use with antipsychotics promotes non-adherence (Sverdrup Efjestad et al., 2017). In contrast, patients treated with multiple antidementia medications have lower rates of non-adherence (Brewer et al., 2013).

Published studies often suffer from limitations such as small sample sizes and short follow-up periods (Brewer et al., 2013; Taipale et al., 2014; Bohlken et al., 2015). As in previous manuscripts (Pedrosa-Naudín et al., 2022; Gutiérrez-Abejón et al., 2023), these deficiencies were addressed by conducting a real-world data study with a 1-year follow-up period in Castile and Leon, a region of Spain with approximately 2.4 million inhabitants (Spanish National Statistics Institute, 2024a). An indirect method based on pharmacy records, which has been widely validated for chronic diseases, was used to measure non-adherence (Culig and Leppée, 2014; Pagès-Puigdemont and Valverde-Merino, 2018; Pedrosa-Naudín et al., 2022; Gutiérrez-Abejón et al., 2023). Given these precedents, the main aim of our study is twofold. First, to estimate the prevalence of non-adherence to antidementia medications by making sociodemographic, pharmacological, and clinical comparisons. Second, to analyze the main predictors of medication non-adherence in the dementia population.

## 2 Materials and methods

### 2.1 Real-world study design

A population-based epidemiologic registry study was conducted in Castile and Leon, Spain, with a population of 2,327,420 inhabitants (Spanish National Statistics Institute, 2024b). The study was designed according to the Strengthening the Reporting of Observational Studies in Epidemiology (STROBE) (von Elm et al., 2008) and the REporting of studies Conducted using Observational Routinely collected health Data for Pharmacoepidemiology (RECORD-PE) (Langan et al., 2018) guidelines.

As a real-world study, the eligible population included all patients with at least one dispensation of antidementia medication between January 1 and 31 December 2022. Only patients with inconsistent medication records were excluded. We obtained data on the prescription and dispensing of medicines reimbursed by the National Health System in Spain. These data are available in CONCYLIA (Castile and Leon Health Council, n. d.), the Pharmacy Information System of Castile and Leon. We were granted access to the CONCYLIA data by the Castile and Leon Health Council, 2023.

Informed consent was not required because patient data are anonymized in CONCYLIA. The study was approved by the Valladolid East Ethics Committee on 21 July 2022 (reference number PI-GR-22–2,844).

### 2.2 The CONCYLIA system

CONCYLIA integrates prescription and dispensing data in our region. Prescription data are collected from the Castile and Leon Electronic Prescription System, which covers all primary care prescriptions. This data source also includes all financed medicines dispensed by the network of 1,685 pharmacies in Castile and Leon (Government of Castile and Leon, 2023). To ensure patient anonymity, the CONCYLIA system uses the Patient Identification Code (PIC). This code also allows the integration of prescription and dispensing data. In addition, CONCYLIA contains other relevant information for conducting pharmacoepidemiologic research, such as sociodemographic and health data, including diagnoses.

Furthermore, the classification of medications and diagnoses in this database is based on the Anatomical Therapeutic Chemical Code (ATC) (WHO Collaborating Centre for Drug Statistics Methodology, 2020) and the International Classification of Diseases-10 (ICD-10) (World Health Organization, 2016), respectively.

### 2.3 Variables

As in previous studies (Pedrosa-Naudín et al., 2022; Gutiérrez-Abejón et al., 2023), an indirect method was used to measure adherence to antidementia medication. This method is based on pharmacy records and uses the Medication Possession Ratio (MPR) as a measure of adherence. MPR was used because it is a widely validated measure for chronic diseases, easy to calculate, and economical (Boulet et al., 2012). However, MPR may sometimes overestimate adherence compared to other more conservative measures (Gelzer et al., 2019).

MPR was calculated as the days of supply during a specified follow-up period (1 year), divided by the number of days from the first dispensing to the end of the follow-up period (Andrade et al., 2006). MPR was estimated for each patient using prescription and dispensing data. Due to the inherent limitations of using real-world data, equivalence between dispensing and consumption was assumed, as in other non-adherence studies conducted by our group (Pedrosa-Naudín et al., 2022; Gutiérrez-Abejón et al., 2023). In addition, MPR was assessed for each of the antidementia medications financed by the Spanish National Health System (ATC codes N06DA02 (donepezil), N06DA03 (rivastigmine), N06DA04 (galantamine), and N06DX01 (memantine)). Finally, MPR was reported as a continuous variable and results were expressed as percentages.

Patients were considered non-adherent if their MPR was less than 80% (Baumgartner et al., 2018; Liu et al., 2022; Pedrosa-Naudín et al., 2022; Gutiérrez-Abejón et al., 2023). In addition, different levels of adherence were identified: null (<20%), poor (20%–49%), moderate (50%–79%) and adherent (>80%) (Krivoy et al., 2016; Pedrosa-Naudín et al., 2022).

Additional sociodemographic and clinical variables were included in the study. Sociodemographic variables included age, sex, institutionalization, and urbanicity. Clinical variables included antidementia medication, comorbidities, multiple prescribers, and polypharmacy.

To assess the evolution of non-adherence across life stages, different age groups were defined: middle-aged adults (<65 years), older adults (65–79 years), and elderly (≥80 years). We have used the variable of urbanicity, considering as urban those localities with 2,500 inhabitants or more.

Comorbidities of interest include cardiovascular and metabolic diseases such as diabetes mellitus, mental illnesses such as depression and/or anxiety and psychotic disorders, and neurodegenerative diseases such as Parkinson’s disease. Diagnoses were identified using ICD-10 (World Health Organization, 2016).

The variable multiple prescribers refers to the prescription of medications to the same patient by three or more different physicians (Green et al., 2007). Finally, polypharmacy was defined based on the concomitant use of five or more medications according to WHO recommendations (WHO, 2019).

### 2.4 Statistical analysis

Descriptive results are presented as prevalence rates or percentages with an associated 95% confidence interval (95% CI), or as means with their associated standard deviation (SD). The Kolmogorov-Smirnov and Shapiro-Wilk tests were used to verify the assumption of the normal distribution of the sample.

Statistical differences between continuous variables were determined using Student’s t-test (t), while differences between categorical variables were assessed using Pearson’s chi-squared test (χ^2^).

A multivariate logistic regression with a forward selection approach was conducted to determine factors associated with non-adherence to antidementia medications. The results were reported as adjusted odds ratios (AOR) with their 95% confidence intervals (95% CI). Variables included in the model were those described previously, including sociodemographic factors (age, sex, institutionalization, and urbanicity) and clinical factors (antidementia drug, pharmacological approach, comorbidities, multiple prescribers, and polypharmacy).

All variables included in the model except age were categorical. No missing values were reported for any of the variables. The absence of collinearity between the variables included in the model was tested using Cohen’s variance inflation factor (VIF).

Statistical analyses were performed with SPSS software (version 25.0, SPSS Inc., Chicago, IL, USA). The significance level was set at *p*-value ≤0.05.

## 3 Results

### 3.1 Baseline characteristics

In 2022, 0.75% of the population of Castile and Leon used at least one antidementia medication. Among the population aged 80 years and older, the prevalence of use increased to 6.12%. Furthermore, the prevalence was almost 2.5 times higher in women than in men (4.35% vs 2.84%; *p* = 0.001).

The baseline characteristics of the population who received antidementia medication are presented in Table 1, categorized by sex. The study population had a mean age of 83.83 ± 7.28 years. Nearly 30% of the population were institutionalized, and over 60% lived in urban areas.

**TABLE 1:** Baseline characteristics of the population using antidementia medications in Castile and Leon (Spain).

	Total	Male	Female	p
N	17,563	5,597	11,966	
Sociodemographic characteristics				
Age (mean ± SD)	83.83 ± 7.28	82.33 ± 7.58	84.54 ± 7.03	0.001
Distribution by age groups (95% CI)				
<65	1.58 (1.4–1.77)	2.43 (2.03–2.83)	1.19 (0.99–1.38)	0.001
65–79	23.66 (23.03–24.29)	29.71 (28.52–30.91)	20.83 (20.11–21.56)	0.001
≥80	74.75 (74.11–75.4)	67.86 (66.63–69.08)	77.98 (77.24–78.72)	0.001
Institutionalized (95% CI)	28.15 (27.48–28.82)	23.01 (21.91–24.12)	30.55 (29.73–31.38)	0.001
Urbanicity (95% CI)	61.71 (60.99–62.43)	62.39 (61.12–63.66)	61.39 (60.52–62.26)	0.216
Clinical characteristics (95% CI)				
Polypharmacy	93.21 (92.84–93.58)	93.26 (92.61–93.92)	93.18 (92.73–93.63)	0.837
Multiple prescribers	68.99 (68.3–69.67)	70.84 (69.65–72.03)	68.12 (67.28–68.95)	0.001
Antidementia medications				
Donepezil	43.49 (42.76–44.23)	43.84 (42.54–45.14)	43.33 (42.44–44.22)	0.522
Rivastigmine	36.84 (36.13–37.55)	37.91 (36.64–39.18)	36.34 (35.47–37.2)	0.044
Memantine	30.7 (30.02–31.38)	28.18 (27–29.35)	31.88 (31.05–32.72)	0.001
Galantamine	3.84 (3.55–4.12)	4.47 (3.93–5.01)	3.54 (3.21–3.87)	0.003
Pharmacological approach				
One medication (AChEI or memantine)	86.67 (86.16–87.17)	87.26 (86.39–88.13)	86.39 (85.77–87)	0.112
Combination (AChEI and memantine)	13.33 (12.83–13.84)	12.74 (11.87–13.61)	13.61 (13–14.23)	0.112
Concomitant diagnostics (95% CI)				
Cardiovascular disease	79.82 (79.22–80.41)	77.85 (76.76–78.93)	80.74 (80.03–81.44)	0.001
Depression and/or anxiety	57.09 (56.36–57.82)	49.15 (47.84–50.46)	60.81 (59.93–61.68)	0.001
Psychotic disorders	38.63 (37.91–39.35)	41.79 (40.5–43.08)	37.16 (36.29–38.02)	0.001
Diabetes Mellitus	23.08 (22.46–23.71)	26.53 (25.38–27.69)	21.47 (20.73–22.2)	0.001
Parkinson’s disease	7.66 (7.26–8.05)	11.42 (10.58–12.25)	5.9 (5.48–6.32)	0.001

Abbreviations: SD, standard deviation, 95% CI, confidence interval, AChEI, acetylcholinesterase inhibitor.

Regarding clinical variables, it was observed that over 90% of patients were polymedicated and almost 70% had multiple prescribers. AChEIs, mainly donepezil (43.49%), followed by rivastigmine (36.84%), were the most used class of antidementia drugs. The only drug in the NMDA receptor antagonist group, memantine, was used by 30.7% of the population. During the study period, 13.33% of the population received combined therapy.

Among the concomitant diagnoses with dementia, cardiovascular disease was observed in nearly 80% of patients, followed by depression and/or anxiety (57.09%), psychotic disorders (38.63%), diabetes mellitus (23.08%), and Parkinson’s disease (7.66%).

### 3.2 Non-adherence to antidementia medications

**FIGURE 1:**
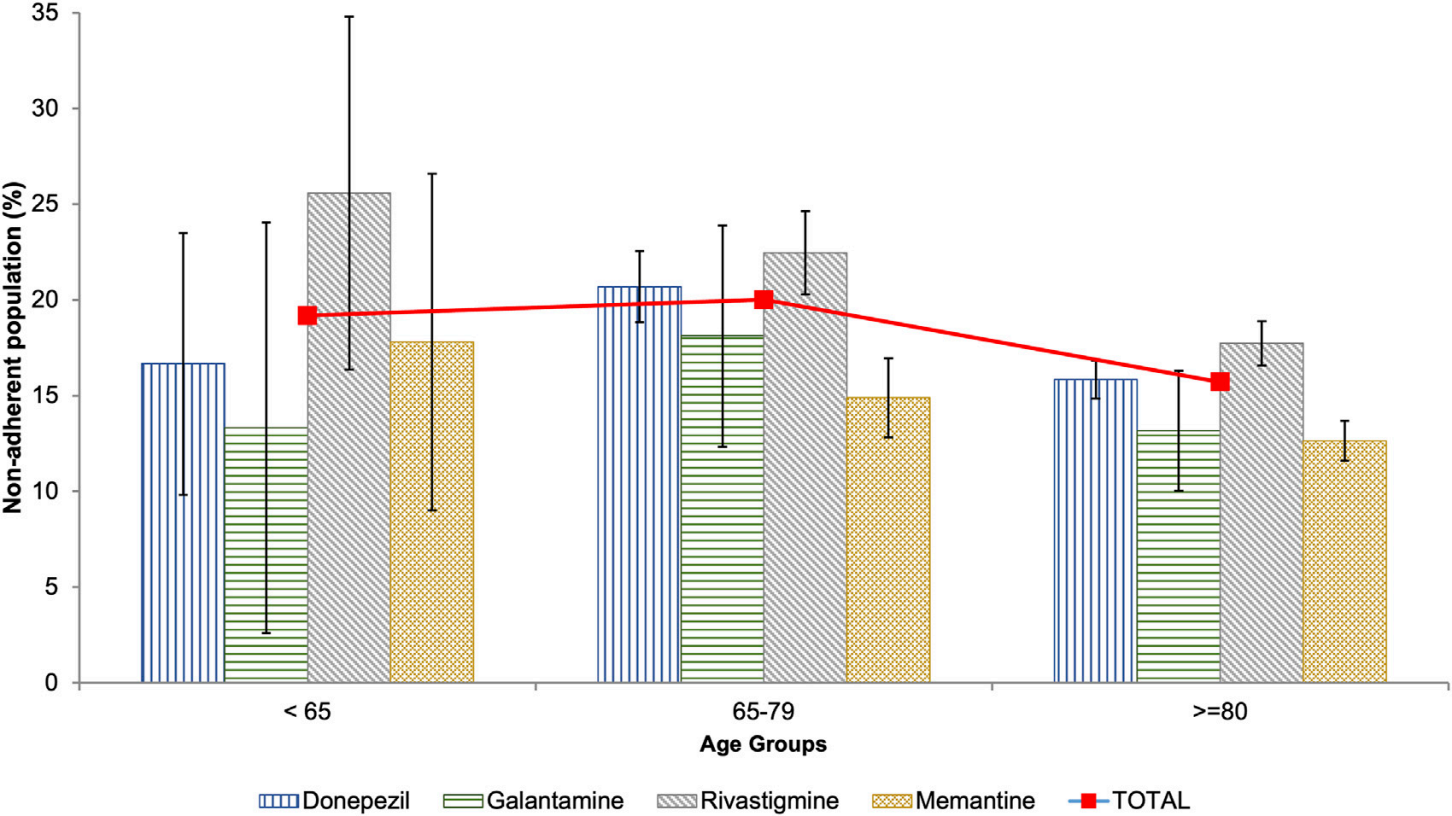
Prevalence of non-adherence to antidementia medication among the population of Castile and Leon (Spain) by age groups.

Non-adherence was found in 16.77% (95% CI: 16.19–17.34) of patients with dementia. Non-adherence to antidementia medication was more prevalent in men than in women (17.94% vs 16.23%; *p* = 0.007). Patients aged 65 to 79 had the highest rate of non-adherence, with a prevalence of 20.01%. This rate decreased to 15.71% in those aged 80 and older (Table 2; Figure 1).

**TABLE 2:** Prevalence of non-adherence to antidementia medications in the population of Castile and Leon (Spain).

		Non-Adherence Prevalence % (95% CI)	p
Sociodemographic characteristics			
Sex			
	Male	17.94 (16.88–18.99)	0.007
	Female	16.23 (15.54–16.92)	
Age groups			
	<65	19.18 (14.25–24.11)	0.001
	65–79	20.01 (18.73–21.29)	
	≥80	15.71 (15.06–16.35)	
Urbanicity			
	Yes	17.1 (16.37–17.84)	0.131
	No	16.19 (15.26–17.11)	
Institutionalized			
	Yes	13.05 (12.09–14.01)	0.001
	No	18.29 (17.58–18.99)	
Clinical characteristics			
Multiple prescribers			
	Yes (≥3 prescribers)	16.99 (16.3–17.69)	0.251
	No (<3 prescribers)	16.26 (15.24–17.29)	
Polypharmacy			
	Yes (≥5 drugs)	16.16 (15.58–16.75)	0.001
	No (<5 drugs)	25.33 (22.71–27.95)	
Antidementia medications			
	Rivastigmine	19.01 (18.01–20.02)	0.001
	Donepezil	17.08 (16.21–17.95)	
	Galantamine	14.51 (11.77–17.25)	
	Memantine	13.23 (12.3–14.16)	
Pharmacological approach			
Number of medications			
One medication (AChEI or memantine)		17.34 (16.76–17.92)	0.001
Combination (AChEI and memantine)		13.29 (12.77–13.82)	
Concomitant diagnostic**s**			
Cardiovascular disease		16.28 (15.64–16.91)	0.001
Diabetes Mellitus		15.55 (14.38–16.71)	
Depression and/or anxiety		15.36 (14.63–16.1)	
Parkinson’s disease		14.99 (12.99–16.99)	
Psychotic disorders		14.99 (14.11–15.87)	

Abbreviations: 95% CI: confidence interval, AChEI, acetylcholinesterase inhibitor

There were no differences in the prevalence of non-adherence between patients who lived in urban areas and those who did not, or between those with multiple prescribers and those without. On the other hand, non-polymedicated patients had a higher prevalence of non-adherence compared to polymedicated patients (25.33% vs 16,16%; *p* = 0.001). Similarly, patients who used only one antidementia medication were more likely to be non-adherent than those who used a combination therapy (17.24% vs 13.29%; *p* = 0.013) (Table 2).

AChEIs were associated with the highest prevalence of non-adherence, especially rivastigmine with 19.01%. On the other hand, memantine, an NMDA receptor antagonist, was associated with the lowest prevalence of non-adherence at 13.23% (Table 2). This pattern is consistent across all age groups, except for patients under 65 years of age, where galantamine has the lowest prevalence of non-adherence (Figure 1).

The prevalence of non-adherence was found to be higher (16.28%) in patients with concomitant cardiovascular disease. In contrast, patients with Parkinson’s disease and psychotic disorders had the lowest rates of non-adherence (Table 2).

Among patients considered non-adherent to antidementia medications, only 1.22% showed complete non-adherence, while 12.67% showed poor adherence and 86% were at moderate adherence levels. The observed pattern was consistent across all characteristics examined, including sociodemographic and clinical factors and concomitant diagnoses (Table 3).

**TABLE 3:** Distribution of population characteristics by level of adherence to antidementia medications in Castile and Leon (Spain).

		Non-adherent			Adherent
Adherence level		None (<20)	Poor (20–49)	Moderate (50–79)	(≥80)
	Total	0.19 (0.13–0.26)	1.98 (1.76–2.2)	13.46 (12.93–13.99)	84.36 (83.8–84.93)
Sociodemographic characteristics (95% CI)					
Sex					
	Male	0.14 (0.04–0.24)	2.04 (1.64–2.43)	14.55 (13.57–15.53)	83.27 (82.24–84.31)
	Female	0.22 (0.13–0.31)	1.95 (1.69–2.21)	12.96 (12.33–13.59)	84.86 (84.19–85.54)
Age group					
	<65	0 (0–0)	2.92 (0.79–5.05)	14.58 (10.12–19.05)	82.5 (77.69–87.31)
	65–79	0.24 (0.08–0.4)	2.21 (1.73–2.68)	16.23 (15.04–17.41)	81.32 (80.07–82.58)
	≥80	0.18 (0.11–0.26)	1.89 (1.65–2.13)	12.58 (11.99–13.18)	85.34 (84.71–85.98)
Institutionalized		0.22 (0.08–0.35)	1.36 (1.03–1.69)	10.59 (9.71–11.48)	87.83 (86.89–88.77)
Urbanicity		0.2 (0.11–0.29)	2.05 (1.77–2.33)	13.63 (12.96–14.31)	84.11 (83.39–84.83)
Clinical characteristics (95% CI)					
Polypharmacy (≥5 drugs)		0.19 (0.12–0.27)	1.88 (1.66–2.09)	12.99 (12.45–13.53)	84.94 (84.36–85.51)
Multiple prescribers (≥3)		0.15 (0.08–0.23)	1.93 (1.67–2.19)	13.73 (13.09–14.37)	84.19 (83.5–84.87)
Antidementia medications					
Rivastigmine		0.16 (0.05–0.26)	1.96 (1.6–2.32)	15.74 (14.8–16.68)	82.15 (81.15–83.14)
Donepezil		0.25 (0.14–0.37)	2.24 (1.89–2.58)	13.39 (12.6–14.19)	84.11 (83.26–84.97)
Galantamine		0.32 (0.12–0.56)	2.07 (0.96–3.19)	11.16 (8.7–13.63)	86.44 (83.76–89.12)
Memantine		0.12 (0.02–0.22)	1.1 (0.81–1.39)	10.98 (10.11–11.85)	87.8 (86.89–88.7)
Pharmacological approach					
One medication (AChEI or memantine)		0.21 (0.14–0.29)	2.17 (1.92–2.41)	13.83 (13.25–14.41)	83.79 (83.17–84.41)
Combination (AChEI and memantine)		0.09 (0.03–0.21)	0.84 (0.46–1.22)	11.25 (9.95–12.55)	87.82 (86.47–89.17)
Concomitant diagnostics (95% CI)					
Cardiovascular disease		0.17 (0.09–0.24)	1.97 (1.73–2.21)	13.04 (12.46–13.63)	84.82 (84.2–85.45)
Diabetes Mellitus		0.14 (0.02–0.25)	1.85 (1.41–2.28)	12.51 (11.44–13.58)	85.51 (84.37–86.65)
Depression and/or anxiety		0.2 (0.11–0.29)	1.91 (1.63–2.19)	12.14 (11.48–12.81)	85.75 (85.03–86.46)
Psychotic disorders		0.19 (0.08–0.3)	1.76 (1.43–2.09)	11.83 (11.03–12.63)	86.22 (85.36–87.07)
Parkinson’s disease		0.41 (0.05–0.78)	1.82 (1.07–2.58)	11.76 (9.95–13.58)	86 (84.04–87.96)

Abbreviations: 95% CI, confidence Interval, AChEI, acetylcholinesterase inhibitor

### 3.3 Risk factor to non-adherence to antidementia medications

The use of rivastigmine (AOR = 1.25) and having multiple prescribers (AOR = 1.15) were found to be associated with an increased likelihood of non-adherence to antidementia medications. On the other hand, certain factors were found to be protective against non-adherence. These included sociodemographic factors such as age (AOR = 0.98) and institutionalization (AOR = 0.75), clinical factors such as polypharmacy (AOR = 0.67), combination therapy (AOR = 0.54) and memantine use (AOR = 0.73), and concomitant diagnoses such as psychotic disorders (AOR = 0.91), depression and/or anxiety (AOR = 0.83), and Parkinson’s disease (AOR = 0.8) (Table 4).

**TABLE 4:** Risk Factors for non-adherence to antidementia medications in Castile and Leon (Spain).

	AOR (95% CI)	p
Sociodemographic characteristics		
Age	0.98 (0.98–0.99)	0.001
Institutionalized	0.75 (0.68–0.83)	0.001
Clinical characteristics		
Multiple prescribers (≥3)	1.15 (1.05–1.26)	0.004
Polypharmacy (≥5)	0.67 (0.58–0.79)	0.001
Antidementia drugs		
Rivastigmine	1.25 (1.14–1.36)	0.001
Memantine	0.73 (0.67–0.81)	0.001
Pharmacological approach		
Combination (AChEIs and NMDARs) (Yes)	0.54 (0.44–0.85)	0.001
Concomitant diagnostics		
Psychotic disorders	0.91 (0.83–0.99)	0.033
Depression and/or anxiety	0.83 (0.76–0.91)	0.001
Parkinson’s disease	0.80 (0.67–0.94)	0.007

Abbreviations: AOR, adjusted Odds Ratio, 95% CI, confidence interval.

## 4 Discussion

In Castile and Leon, Spain, 6% of the population over 80 years old use medication for dementia. The prevalence of medication use is more than twice as high in women as in men. In 2022, approximately one in six patients were non-adherent to antidementia medications, particularly rivastigmine with one in five patients. The study found that male patients and non-institutionalized patients had poorer results. On the other hand, a decrease in the prevalence of non-adherence was observed after the age of 80. Multiple prescribers and the use of rivastigmine were identified as predisposing factors for non-adherence.

The prevalence of antidementia medication use was 0.75% in the general population and 6% in the population over 80 years of age. These findings are comparable to a study conducted in the United Kingdom (Donegan et al., 2017). Nevertheless, the prevalence rate differs from other European countries, being higher than in Hungary (0.09%) (Balázs et al., 2022), but lower than in Austria, where the prevalence rate is 1% in the general population and 11% in the population over 80 years of age (Wurm et al., 2020). Considering the prevalence of dementia in Spain (0.05%–39.2% across age groups) (Spanish Ministry of Health, 2019), it is noteworthy that the use of antidementia medications in the affected population is significantly low. However, the proportion of patients diagnosed with dementia who receive medication varies widely in the literature, ranging from 14% to 70% (Koller et al., 2016; Calvó-Perxas et al., 2017; Balázs et al., 2022).

AChEIs, indicated for mild to moderate disease (Hort et al., 2010), are the most used antidementia drugs, especially donepezil. This pattern has also been observed in other studies conducted in Spain (Calvó-Perxas et al., 2017) and Europe (Taipale et al., 2014; Bohlken et al., 2015; Donegan et al., 2017; Wurm et al., 2020). An exception is the use of galantamine, which was significantly lower than in other studies (Taipale et al., 2014; Bohlken et al., 2015; Wurm et al., 2020). In this sense, galantamine has been shown to reduce the incidence of depressive spectrum disorders and is associated with a reduction in caregiver burden (Scott and Goa, 2000). However, the use of galantamine has been associated with the development of cardiac conduction disorders (Fisher and Davis, 2008) and with an increase in cardiovascular mortality (Spanish Agency for Medicines and Health Products, 2005).

On the other hand, one-third of the patients used memantine, which is indicated for more advanced stages of the disease (Wong, 2016). This finding is consistent with other studies (Taipale et al., 2014; Bohlken et al., 2015). Memantine is commonly used due to its multiple actions as an NMDA receptor antagonist, antiparkinsonian, antidepressant, and neuroprotective and dopaminergic agent (Johnson and Kotermanski, 2006).

As in other studies (Taipale et al., 2014; Calvó-Perxas et al., 2017; Scuteri et al., 2021), the combined use of antidementia medicines was common in our region. During the study period, nearly 15% of patients used more than one antidementia medication. The combination of an AChEI and an NMDA receptor antagonist produces synergistic effects that delay cognitive and functional decline.

Several studies (Mucha et al., 2008; Blais et al., 2009; Poon et al., 2009; Borah et al., 2010; Le Couteur et al., 2012; Haider et al., 2014; Kim et al., 2018; Ku et al., 2018; Kongpakwattana et al., 2019) have used MPR to measure non-adherence to antidementia medications. The prevalence of non-adherent patients in these studies ranges from 6% (Blais et al., 2009) to 42% (Borah et al., 2010). Our findings indicate that approximately 17% of patients are non-adherent, falling in the 50th percentile of this range. However, interpreting the difference between observed results requires caution due to variations in patient sample sizes and MPR cut-off point for determining non-adherence.

The non-adherence rate varied among the antidementia medications, with AChEIs showing a higher rate compared to memantine. Specifically, rivastigmine had a non-adherence rate of nearly 20%, which is 6% higher than memantine. These findings are consistent with other studies that have shown rivastigmine and donepezil to be the least adherent drugs (Bohlken et al., 2015; Wurm et al., 2020; Balázs et al., 2022). This is likely due to the worse side effect profile of AChEIs compared to memantine (Birks, 2006). Some studies have suggested that patients who used rivastigmine patches had better adherence than those who used capsules (Molinuevo and Arranz, 2012; Balázs et al., 2022), while other studies did not find this improvement (Chang et al., 2019). Because of these conflicting results, our study evaluated the rivastigmine formulations together.

Historically, the combined use of AChEI and memantine has been associated with higher compliance and persistence rates compared to monotherapy (Brewer et al., 2013; Yu et al., 2020), particularly in patients initiating treatment (Kongpakwattana et al., 2019). In this sense, our study confirmed that patients on monotherapy had a 25% higher rate of non-adherence compared to those on combination therapy.

Similar to previous studies (Maxwell et al., 2014; Ofori-Asenso et al., 2018), switching from one AChEI to another within the same group was infrequent. Additionally, designating switchers as non-adherent patients may be a controversial issue (Gardette et al., 2014). Therefore, like other authors (Mucha et al., 2008; Brewer et al., 2013), we did not conduct a separate analysis on switching.

Several studies (Steininger and Kostev, 2020; Lu et al., 2021; Olchanski et al., 2023) have observed that non-adherence to AChEIs treatment increases with age. In contrast, other studies have reported lower rates of non-adherence (Borah et al., 2010) and non-persistence (Amuah et al., 2010) among older patients. Our study found that non-adherence rates were highest among patients under the age of 80, especially those aged 65 to 79. This is because older patients are more likely to have a caregiver who can monitor their medication intake, leading to improved adherence (Small and Dubois, 2007). In our region, three out of 10 patients with dementia reside in nursing homes, so the presence of caregivers is a key issue. Female sex is a factor classically associated with non-adherence to antidementia medications (Kröger et al., 2010; Maxwell et al., 2014; Taipale et al., 2014; Bohlken et al., 2015). This association is because women are more hesitant to have a caregiver than men (Small and Dubois, 2007). In our study, the rate of non-adherence is slightly lower in women than in men. The results were not surprising, as the presence of a caregiver is strongly associated with female sex, as one in three women with dementia are institutionalized, compared to one in five men. Dementia is a frequent cause of institutionalization (Luppa et al., 2010), with nursing home admission rates ranging from 30%–50% of patients (Schulze et al., 2015). Consistent with previous research institutionalized patients had a lower prevalence of non-adherence compared to non-institutionalized patients (Abughosh and Kogut, 2008; Gadzhanova et al., 2010; Pedrosa-Naudín et al., 2022). Certainly, supervision of medication intake is a factor that promotes adherence (Algameel, 2020). No differences were found in the prevalence of non-adherence associated with living in urban or rural areas, confirming the results of another study on this class of medication (Bohlken et al., 2015). Limited evidence exists on the impact of the number of prescribers on medication adherence, with conflicting results (Green et al., 2007; Hansen et al., 2015; Pedrosa-Naudín et al., 2022). Pedrosa et al. (Pedrosa-Naudín et al., 2022) observed that patients with three or more prescribers had a lower prevalence of non-adherence to antidepressants. In contrast, Hansen et al. (Hansen et al., 2015) found the opposite for antihypertensives and hypolipidemic agents. No previous studies have assessed the influence of this variable on patients with dementia.

Previous studies have reported that patients with dementia have high rates of polypharmacy, ranging from 55% to 72% (Kongpakwattana et al., 2019; Growdon et al., 2021). In our region, the rate of polypharmacy was found to be over 90%. This high rate may be attributed to the advanced age of the population and the presence of comorbidities. Furthermore, elderly patients with dementia who are polymedicated are at a higher risk of using potentially inappropriate medication (PIM) compared to those who are not (Lau et al., 2010). PIMs identified by the Beers criteria for dementia, such as anticholinergics, benzodiazepines, and H_2_ receptor antagonists, require special attention due to their side effects on the central nervous system (Gray et al., 2015; By the 2023 American Geriatrics Society Beers Criteria^®^ American Geriatrics Society Beers Criteria^®^ Update Expert Panel, 2023).

Polypharmacy may influence the prevalence of non-adherence, particularly in elderly patients aged 85 years and older (Onder et al., 2020). In the case of antidementia medications, some studies suggest that polypharmacy may contribute to non-adherence (Marcum and Gellad, 2012; Booker et al., 2016), while others have not found evidence to support this claim (Amuah et al., 2010; Bohlken et al., 2015). Our study found that non-adherence prevalence was 10% lower in polymedicated patients than in non-polymedicated patients. Similar behavior has been observed in Spain when evaluating adherence to other psychotropic medications (Serna et al., 2015; Pedrosa-Naudín et al., 2022). In this sense, polymedicated patients often have multiple comorbidities that require more detailed monitoring by the physician (Herrmann et al., 2007). Furthermore, polymedication is strongly associated with institutionalization in our region, which may explain these findings.

Some published results (Kröger et al., 2010; Brady and Weinman, 2013) suggest that comorbidities are associated with a higher prevalence of non-adherence in patients with dementia. However, this hypothesis has not been confirmed in other studies (Taipale et al., 2014; Bohlken et al., 2015). Our study did not assess the overall impact of comorbidity on non-adherence. Instead, we examined the effect of each disease individually. Parkinson’s disease, depression and/or anxiety, as well as psychotic disorders, have been identified as protective factors against non-adherence. On the other hand, patients with cardiovascular disease and diabetes mellitus had a higher prevalence of non-adherence. The higher likelihood of institutionalization among patients with mental illness compared to those with cardiometabolic disease (37% vs 33%) may explain these findings.

The use of antipsychotics in patients with dementia is a sensitive issue because it is associated with increased mortality (Maust et al., 2015). Several international clinical guidelines recommend reduce use, although results have been mixed (Valiyeva et al., 2008; Gallini et al., 2014; Maust et al., 2015; Gerlach et al., 2021; Rantsi et al., 2023). In general, it is recommended to use antipsychotics at low doses for a short period of up to 6 weeks, and only after non-pharmacological therapies have been unsuccessful (Watt et al., 2017). In our study we observed two patterns related to the use of antipsychotics. First the prevalence of psychotic disorders was higher than in other studies, possibly due to the influence of the COVID-19 pandemic (Campitelli et al., 2021). Second, in contrast to other study, we found that antipsychotic use was a protective factor against non-adherence. This pattern may be due to the fact that the rate of antipsychotic use is twice as high in institutionalized patients compared to non-institutionalized patients.

The study’s main strength is its use of real-world data, including a large sample size of over 17,000 patients with dementia and a long follow-up period. This study design allowed us to overcome certain limitations of previous research (Brewer et al., 2013; Taipale et al., 2014; Bohlken et al., 2015).

Finally, it is important to mention other limitations in addition to the methodological ones inherent in this type of study (Suissa and Garbe, 2007). An indirect method based on pharmacy records was used to measure non-adherence, assuming that dispensing is equivalent to taking medication. In this sense, our team has previously used this method in investigations with successful results (Pedrosa-Naudín et al., 2022; Gutiérrez-Abejón et al., 2023). It has been observed that the use of MPR may overestimate adhesion because it does not account for gaps between refills (Le Couteur et al., 2012). In addition, a patient was considered non-adherent if MPR was less than 80%. This cut-off point, although previously used (Pedrosa-Naudín et al., 2022; Gutiérrez-Abejón et al., 2023), is not universal (Baumgartner et al., 2018). However, MPR has been commonly used as a measure of adherence in chronic diseases (Boulet et al., 2012). Furthermore, MPR is one of the most effective methods for measuring adherence in patients who use psychotropic medication (Karve et al., 2009). On the other hand, CONCYLIA lacks data on medication usage in hospitals and private medical practices. This limitation is irrelevant since over 95% of the population of Castile and Leon, is covered by the public health system (Spanish National Statistics Institute, 2024a), which finances antidementia medication. Finally, there is no data on other drugs that may improve survival in some patients with dementia (Wu et al., 2015), such as piracetam, ginkgo biloba, or ergot derivatives. Some of these medications have been shown to be equally effective against dementia symptoms, such as ginkgo biloba (Mazza et al., 2006), or to have neuroprotective effects, such as piracetam (Fang et al., 2014). As a result, the use of global antidementia medications may be underestimated. In conclusion, our study provides new insights into the traditional profile of non-adherent patients to antidementia medications. This profile is associated with three primary characteristics: advanced age, female sex, and the use of antipsychotic medications (Maxwell et al., 2014; Bohlken et al., 2015; Sverdrup Efjestad et al., 2017). In Castile and Leon, Spain, younger male patients with cardiometabolic disease are more likely to be non-adherent to antidementia medications. The influence of a higher institutionalization rate among women was crucial for this observed change in behavior.

A study was conducted with real-world data, using an indirect method to measure adherence because the large number of patients did not allow the use of a more accurate direct method (Culig and Leppée, 2014). Our group’s previous experience in similar studies allows us to confirm that MPR is a robust indicator for identifying non-adherent patients. Indeed, this indicator is currently used in the regional public health system for two purposes.

First, CONCYLIA automatically identifies patients with an MPR <80 as possible non-adherent patients. Subsequently, CONCYLIA automatically generates a report, which is transmitted electronically to the healthcare professional responsible for the patient. The healthcare professional, such as a physician, pharmacist, or nurse, verifies whether the patient is really non-adherent. In positive cases, the patient is contacted to reinforce the importance of adherence in the success of pharmacological treatment (Osterberg and Blaschke, 2005). Secondly, it has enabled the identification of subpopulations that may be sensitive to the consequences of non-adherence and therefore require closer monitoring by healthcare professionals (The Association of British Pharmaceutical Industry, 2011). Of note among these subpopulations are young male adults who use rivastigmine and have concomitant cardiometabolic disease. An important case study is the institutionalized population. The presence of a caregiver for medication monitoring in institutionalized patients is a clear protective factor against non-adherence (Algameel, 2020). However, it is still concerning that even 10% of institutionalized patients experience non-adherence to antidementia medications. This situation could be an important therapeutic issue, considering that patients admitted to nursing homes are more frail and have more advanced stages of dementia (Cross et al., 2024) To improve our understanding of this issue, future studies should be conducted with smaller cohorts. Additionally, some PIMs are associated with a cognition-impairing effect that exacerbates the disease (Park et al., 2017), so it is important to review patients’ pharmacotherapy plans based on Beers (American Geriatrics Society Beers Criteria^®^ Update Expert Panel, 2023) or STOPP/START (O’Mahony et al., 2023) criteria.

## Data Availability

Restrictions apply to the availability of these data. Data were obtained from regional health authorities [Gerencia Regional de Salud (GRS)] and may be requested from conciertofco@saludcastillayleon.es.
